# Ultralong-Lived Up-Conversional Room-Temperature Afterglow Materials with a Polyvinyl Alcohol Substrate

**DOI:** 10.3390/polym14122414

**Published:** 2022-06-14

**Authors:** Lulu Zhou, Bin Wu, Ben Shi, Xinyan Zhu, Shen Shen, Liangliang Zhu

**Affiliations:** 1 State Key Laboratory of Molecular Engineering of Polymers, Department of Macromolecular Science, Fudan University, Shanghai 200438, China; 18110440017@fudan.edu.cn (L.Z.); 20110440047@fudan.edu.cn (S.S.); 2 State Key Laboratory of Molecular Engineering of Polymers, Department of Chemistry, Fudan University, Shanghai 200433, China; 16110440032@fudan.edu.cn (B.W.); shi_ben@fudan.edu.cn (B.S.); 18210220020@fudan.edu.cn (X.Z.)

**Keywords:** room-temperature afterglow, photoluminescent lifetime, doping, smearing, up-conversional

## Abstract

Room-temperature afterglow (RTA) materials have a wide range of applications in imaging, lighting, and therapy, due to their long lifetime and persistent luminescence after the light source is removed. Additionally, near-infrared light with low energy and a high penetration rate ensures its irreplaceable importance in imaging and therapy. Thus, it is vital to design RTA materials excited by NIR. In the present study, we select up-conversion nanoparticles (UCNPs) as the donor and add them into hybrids, obtained by dispersing coronene tetra-carboxylate salt (CS) into a polyvinyl alcohol (PVA)-substrate through a series of mixing methods. Through radiation energy transfer between the donor UCNPs and the acceptor CS, a kind of RTA film with a photoluminescence lifetime of more than 2 s under NIR excitation was successfully achieved, and these films could maintain persistent naked-eye-distinguishable luminescence after withdrawing the excitation light source. Furthermore, the films obtained from UCNP doping into CS/PVA hybrids were found to exhibit better RTA performance than those from smearing. This idea of up-conversion afterglow broadens the tuning and application scope for polymer-based luminescent materials.

## 1. Introduction

Room-temperature afterglow (RTA) materials refer to those that can continuously emit light for a period of time at room temperature when the external excitation light source is removed [1,2,3,4,5,6]. In 1996, Matsuzawa found that by doping rare-earth ions, europium (Eu) and dysprosium (Dy), into strontium aluminate (SrAl_2_O_4_), an inorganic afterglow material with a good emission for 10 h could be obtained [7]. Subsequently, this inorganic afterglow material has been widely used in various luminescence fields, such as lighting, anti-counterfeiting, and biological imaging [8,9,10]. DC Sheppeck et al. achieved continuous lighting for minutes or even hours after the lamp was turned off by adding afterglow luminescent powder to electroluminescent lamps [10]. Nevertheless, the preparation methods of inorganic RTA materials usually need harsh conditions [7,11]. Moreover, most materials need to be ground into powder before use, which greatly limits the application field of inorganic afterglow materials [12,13,14]. Therefore, the research on flexible organic afterglow materials is an urgent demand [6,15,16,17].

As for the organic afterglow, when the molecule is excited, the ground state S_0_ can be transformed into the S_1_ excited state, and then a large number of S_1_ states are transformed into T_1_ states through an extremely high ratio of intersystem crossing (ISC) compared to ordinary phosphorescence. Thus, the persistent afterglow phosphorescence can be observed with naked eyes, while the T_1_-state excitons are transferred back to S_0_ [6,18,19]. However, it is particularly difficult to achieve RTA in pure organic materials, due to the low probability of intersystem crossing (ISC) between S_1_ and T_1_, and also the existence of non-radiative transitions caused by vibration and oxygen quenching [19,20,21,22,23]. Therefore, doping organic molecules into polymer substrates, such as polymethyl methacrylate (PMMA) or polyvinyl alcohol (PVA) [24,25,26,27], and utilizing the rigid environment provided by polymers to minimize non-radiative transitions of organic molecules, has gradually become a simple and efficient method to realize RTA organic materials. To be exact, the RTA emission mainly comes from doped organic molecule phosphors, while the polymers only provide a rigid environment.

PVA as a good oxygen barrier with high tensile strength and flexibility has become a superior matrix for carrying phosphorescent chromophores to minimize vibration and oxygen quenching in the triplet state of the phosphor [26,28]. In this way, the intramolecular ISC ratio of the chromophore can be improved, thereby enabling the RTA of the system. Suman K. and his partners mixed coronene tetra-carboxylate salt (CS) into the PVA substrate to realize a greenish-yellow organic RTA material [25]. On this basis, a tunable RTA emission was successfully achieved by using CS as the donor and other organic chromophores as the acceptors in subsequent studies. However, the current regulation of CS/PVA RTA material is still subjected to UV excitation [25,27], which is relatively harmful to the human body and limits the further application of this system due to the high energy and weak penetrating ability of UV light. It is known that near-infrared (NIR) has been widely used in the fields of anti-counterfeiting and biological imaging or therapy because of its lower energy and higher penetration [29,30,31]. Nevertheless, there are relatively few organic materials with a μs-ms lifetime that use NIR excitation to achieve RTA, and it is necessary to fabricate longer lifetime NIR-excited RTA materials [29,32,33].

**Figure 1:**
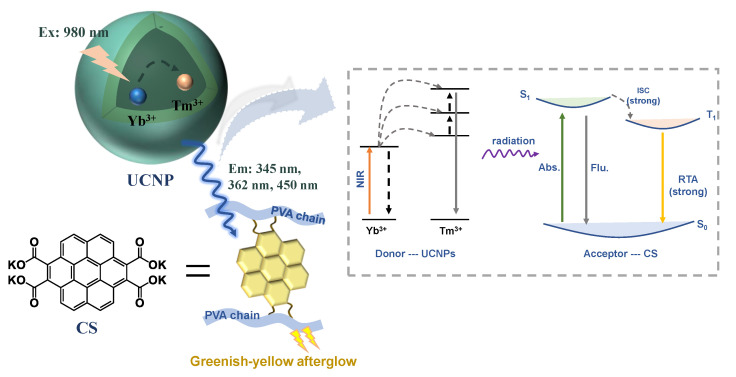
Simplified illustration of the radiation energy transfer between donor UCNPs and acceptor CS under NIR excitation. (Abs. = absorbance, Flu. = fluorescence, ISC = intersystem crossing).

Herein, an organic RTA system (Figure 1) with a lifetime of more than 2 s under NIR excitation was successfully realized by introducing up-conversion nanoparticles (UCNPs) excited by 980 nm laser as the donor [34,35], which can convert NIR light into UV light to activate the acceptor CS to exhibit a greenish-yellow afterglow when mixed into a PVA substrate (Table 1). In this work, two kinds of UCNPs (water-soluble UCNPs and oil-soluble UCNPs) were introduced to the CS/PVA hybrids by doping and smearing, respectively. By comparing the RTA lifetime of the systems with UCNPs added in different mixing ways, the doping is proven to be better than the smearing method in the RTA performance, due to the higher energy transfer efficiency. It is undeniable that stable RTA materials with a lifetime of more than 2 s under NIR excitation were successfully achieved by introducing 980 nm excited UCNPs into the CS/PVA hybrids, which play a crucial role in broadening their application fields.

## 2. Results and Discussion

**Figure 2:**
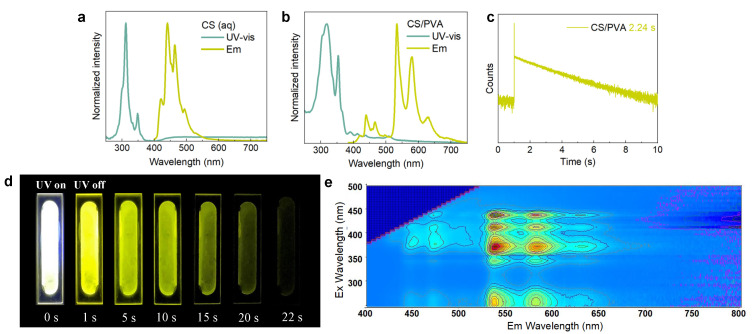
(**a**) Normalized UV-Vis spectrum and photoluminescence spectra of CS in aqueous solution (λ_exc._ = 365 nm); (**b**) normalized UV-Vis spectrum and photoluminescence spectra (λ_exc._ = 365 nm) of CS/PVA hybrids; (**c**) RTA phosphorescence lifetime curve of CS/PVA hybrids (λ_exc._ = 365 nm, λ_monitor_ = 540 nm); (**d**) photographs of the long-lasting luminescence of the CS/PVA film with the UV light excitation on and off; (**e**) excitation–emission mapping of CS/PVA hybrids (λ_exc._ = 250–500 nm, λ_em._ = 400–800 nm).

CS is a kind of light yellow solid powder with excellent water solubility, which presents a yellow transparent solution after dissolving in water [36,37] (see Scheme S1 and Figures S1 and S2 for the complete synthetic procedure). In a solid state, there is no fluorescence generated under UV light irradiation (Figure S3a); whereas, in solution, blue fluorescence appears when irradiated with UV light, but disappears instantly as the light source is withdrawn (Figure S3b, Video S1). The UV-Vis spectrum shows CS has an absorption at 260–370 nm, and the highest point of the emission spectrum is located at 440 nm and 460 nm (Figure 2a). The photoluminescence lifetime of CS at 460 nm is 13.18 ns (Figure S4a), and there is no existence of phosphorescence that has been detected (Figure S4b) while under the excitation of UV light. However, a greenish-yellow RTA material with a strong emission between 515–600 nm and a weaker emission between 400–500 nm could be obtained under the excitation of UV light (Figure 2b) when CS was uniformly dispersed in PVA substrate, of which the lifetime is 2.24 s at 540 nm (Figure 2c). Thus, CS/PVA would show a strong emission in the dark while excited with UV light, and this luminescence could still be maintained for about 22 s after the light source is withdrawn (Figure 2d, Video S2). However, there is no emission that can be observed from the CS/PVA film while under the excitation of 980 nm (Figure S5). The excitation–phosphorescence mapping showed this CS/PVA hybrid can be excited by all the 254 nm, 375 nm, 415 nm, and 445 nm light sources (Figure 2e). Therefore, we hope that by introducing 980 nm excited UCNPs with emission bands covering these wavelengths as energy donors, CS/PVA hybrids can realize NIR-excited RTA by absorbing the light emitted from UCNPs.

NaYF_4_: Yb, Tm UCNPs prepared by a simple thermal decomposition were selected here, and the water-soluble UCNPs could be obtained after the oil-soluble UCNPs were hydrophilized (see complete synthesis procedures in the Supplementary Materials). The water-soluble and oil-soluble UCNPs all exhibited a standard regular hexagonal crystal structure with average particle sizes of 80 nm and 10 nm, respectively (Figure S6) under transmission electron microscopy (TEM), which were basically consistent with the dynamic light scattering (DLS) data (Figure S7). Under the excitation of 980 nm, the emission peaks of these two UCNPs are located at 345 nm, 362 nm, 450 nm, 480 nm, and 650 nm (Figures S8a and S9a), which just cover the excitation wavelengths of the CS/PVA film mentioned above. Subsequently, the lifetime of the emission peaks of these two UCNPs was detected under 980 nm, all of which were within 1 ms (Figures S8b and S9b).

**Figure 3:**
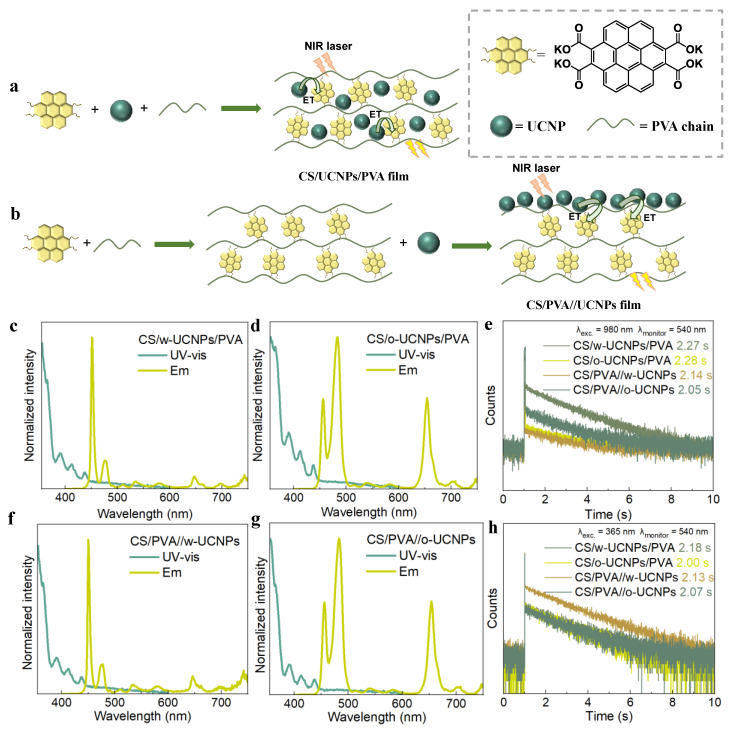
(**a**) Schematic diagram of UCNPs entering CS/PVA hybrid by doping to prepare CS/UCNPs/PVA film; (**b**) schematic diagram of UCNPs entering CS/PVA hybrid by smearing to prepare CS /PVA//UCNPs film; (**c**) normalized UV-Vis spectrum and photoluminescence spectra (λ_exc._ = 980 nm) of CS/w-UCNPs/PVA film; (**d**) normalized UV-Vis spectrum and photoluminescence spectra (λ_exc._ = 980 nm) of CS/o-UCNPs/PVA film; (**e**) comparison of lifetime curves of four films under 980 nm excitation; (**f**) normalized UV-Vis spectrum and photoluminescence spectra (λ_exc._ = 980 nm) of CS /PVA//w-UCNPs film; (**g**) normalized UV-Vis spectrum and photoluminescence spectra (λ_exc._ = 980 nm) of CS /PVA//o-UCNPs film; (**h**) comparison of lifetime curves of four films under 365 nm excitation. (ET = energy transfer).

In terms of the doping method, UCNPs and CS are uniformly incorporated into the PVA film, which is expressed as CS/UCNPs/PVA below (Figure 3a). The water-soluble UCNPs and CS were simultaneously added to the PVA stock solution to prepare the CS/w-UCNPs/PVA film (Figure 3a) (see Supplementary Materials for the complete synthetic procedure), whose emission spectrum under 980 nm demonstrated that UCNPs were mixed into the system and successfully excited the CS/PVA hybrids with an emission range between 515–600 nm. The UV-Vis spectrum showed a strong absorption between 350–450 nm of this film (Figure 3c). As expected, the CS/w-UCNPs/PVA film exhibited a lifetime of 2.27 s under 980 nm excitation (λ_monitor_ = 540 nm) (Figure 3e), which indicated that the CS/PVA hybrids successfully achieved RTA under NIR excitation following the introduction of water-soluble UCNPs by doping, with little effect on its lifetime. The emission spectra of the CS/o-UCNPs/PVA film, which was prepared by doping the oil-soluble UCNPs to the CS/PVA hybrids (see Supplementary Materials for the complete synthetic procedure), also investigated that the UCNPs have excited the CS/PVA hybrids with a 515–600 nm emission band (Figure 3d). Similar to the CS/w-UCNPs/PVA film, this CS/o-UCNPs/PVA film also showed strong absorption between 350–450 nm (Figure 3d) and a 2.28 s lifetime (λ_monitor_ = 540 nm) under 980 nm excitation (Figure 3e). The reason why these films prepared with doping UCNPs maintained good RTA properties is mainly attributed to the well contact between donor UCNPs and acceptor CS inside the PVA substrate through constant high-temperature stirring, which ensured the maximum energy transfer efficiency between them. Furthermore, the greenish-yellow afterglow could still be observed with naked eyes in the dark when the CS/w-UCNPs/PVA and CS/o-UCNPs/PVA films were exposed to UV light excitation (Figure S10, Videos S3 and S4), and then we tested the lifetime (λ_monitor_ = 540 nm) of them under 365 nm. The results display that the addition of water-soluble and oil-soluble UCNPs reduces the RTA lifetime of the systems by 60 ms and 240 ms, respectively (Figure 3h), which is caused by the partial destruction of the hydrogen bonds inside the CS/PVA hybrids. The incompatibility between oil-soluble UCNPs and PVA results in a greater degree of hydrogen-bond destruction inside CS/PVA hybrids, which caused a higher reduction in the lifetime of the system.

For smearing, UCNPs were evenly spread on the surface of the prepared CS/PVA film (Figure 3b), which is stated as CS/PVA//UCNPs below. CS/PVA//w-UCNPs and CS/PVA//o-UCNPs films with absorptions at 350–450 nm (Figure 3f,g) were prepared after water-soluble and oil-soluble UCNPs were added to the CS/PVA hybrids by uniform smearing (Figure 3b) (see Supplementary Materials for the complete synthetic procedure). Both of them also showed RTA phenomenon under the excitation of 980 nm. From the emission (Figure 3f,g) and lifetime spectra (Figure 3e), we can observe that the water-soluble and oil-soluble UCNPs smeared on the film surface have successfully excited the CS/PVA hybrids under the 980 nm laser with lifetimes of 2.14 s and 2.05 s, respectively. The reduced lifetimes of both films relative to the doping method were mainly due to the fact that applying UCNPs to the surface of the CS/PVA film will make the contact between UCNPs and CS not uniform enough. Additionally, compared to the doping method, a farther distance between donor UCNPs and acceptor CS appeared, which certainly reduced the efficiency of energy transfer between them. Moreover, a greater impact on the lifetime of the CS/PVA//o-UCNPs film (reduced by 200 ms) is exhibited than that of the CS/PVA//w-UCNPs film (reduced by 100 ms), since the oil-soluble UCNP has an inevitable drawback of poor compatibility with the PVA substrate (Figure 3e). In addition, the RTA performance of these films under UV light is similar to that of the doping method (Figure S11, Videos S5 and S6), showing a trend of decreasing lifetime (Figure 3h). After obtaining these four films by different mixing methods, the emission intensity of 500–620 nm under UV and NIR excitations was detected, respectively, to compare the afterglow intensity before and after up-conversion. The results show that the addition of UCNPs did not significantly affect the emission intensity of CS/PVA hybrids under UV light (Figure S12a). However, when exposed to near-infrared light, the introduction of oil-soluble UCNPs makes the system exhibit a relatively stronger emission, which is caused by a higher emission of oil-soluble UCNPs than that of water-soluble UCNPs when excited by NIR light (Figure S12b). The quantum yield of oil-soluble UCNPs was 0.16% (IR26 = 0.05% as reference) (Figure S13), and the CS/PVA hybrids also reported with a quantum yield of 23.4% [25]. Therefore, the QY of the up-conversion CS/UCNPs/PVA films will not exceed 0.04%, according to the fact that the QY of the system will not exceed the product of the quantum yields of its individual components.

Briefly, the addition of these two kinds of UCNPs to CS/PVA hybrids, either doped or smeared, can induce RTA under NIR excitation with lifetimes exceeding 2 s. As far as the addition methods of UCNPs are concerned, when the UCNPs is evenly spread on the surface of the CS/PVA hybrids by smearing, the distance between the donor and the acceptor is further, which consequently causes a reduction in the energy transfer efficiency. Moreover, compared with water-soluble UCNPs, when oil-soluble UCNPs were spread on the surface of PVA, they produced greater damage to the RTA performance of the system due to its poor compatibility with PVA. In conclusion, a longer lifetime and more stable RTA materials can be obtained when UCNPs are introduced to the CS/PVA hybrids by a doping method with a maximum energy transfer efficiency.

**Figure 4:**
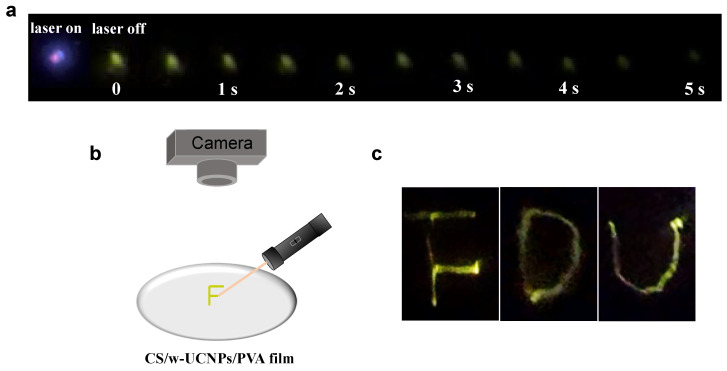
(**a**) Photographs of the long-lasting luminescence of the CS/w-UCNPs/PVA film with the 980 nm laser excitation on and off; (**b**) schematic illustration of laser writing; (**c**) photographs of laser writing of capital letters “F”, “D”, and “U”.

Herein, afterglow hybrid systems with lifetimes up to 2 s under NIR excitation were successfully prepared after adding UCNPs to the CS/PVA films, which offered possibilities for optical anticounterfeiting signatures with the polymer-based afterglow materials. Considering the operability, convenience, and safety, we used a low-power 980 nm laser pointer in the application for experiments. The CS/w-UCNPs/PVA film with an excellent, long afterglow performance was taken as an example here; a 5 s greenish-yellow afterglow could still be observed on this film under the 980 nm laser pointer excitation (Figure 4a). Thus, by selecting the CS/w-UCNPs/PVA film as the “paper”, the 980 nm laser pointer as the “signature pen”, and the greenish-yellow afterglow as the “ink” (Figure 4b), we can successfully write visible patterns, such as capital letters “F”, “D”, and “U”, on the CS/w-UCNPs/PVA film without any trace (Figure 4c, Video S7).

## 3. Conclusions

In general, both water-soluble and oil-soluble NIR-excited UCNPs can realize RTA materials with a lifetime of more than 2 s under NIR excitation when added into CS/PVA hybrids. However, the effects on the final RTA materials were somewhat varied when these two types of UCNPs were added to the CS/PVA mixture using different mixing methods The CS/UCNPs/PVA films obtained by doping had almost no influence on the RTA lifetime under NIR excitation, which were basically the same as those of CS/PVA hybrids under UV light. However, when the CS/PVA//UCNPs was obtained by the smearing method, a long distance and poor contact between the donor and acceptor in the system decreased the energy transfer efficiency, leading to the reduction in RTA lifetime, especially when oil-soluble UCNPs were smeared on the PVA film due to the poor compatibility between them. After fundamentally realizing the near-infrared excited afterglow of polymer-matrix materials, we will make persistent efforts to achieve biological imaging with them in the future. In the end, the preparation method of NIR-excited RTA materials proposed in this paper provides a convenient and universal up-conversion idea for other organic RTA materials with polymer substrates, which is of great significance for broadening their applications in imaging or anti-counterfeiting.

## Figures and Tables

**Table 1:** A list of abbreviation instructions in the study.

Abbreviations	Full Name	Abbreviations	Full Name
RTA	Room-temperature afterglow	UV light	Ultraviolet light
ISC	Intersystem crossing	NIR	Near infrared
S_0_	The ground state	CS/PVA	Coronene tetra-carboxylate salt uniformly dispersed in the PVA matrix
S_1_	The singlet state	w-UCNPs	Water-soluble up-conversion nanoparticles
T_1_	The triplet state	o-UCNPs	Oil-soluble up-conversion nanoparticles
PMMA	Polymethyl methacrylate	CS/UCNPs/PVA	UCNPs mixed with CS/PVA hybrids by doping method
PVA	Polyvinyl alcohol	CS/PVA//UCNPs	UCNPs mixed with CS/PVA hybrids by smearing method
CS	Coronene tetra-carboxylate salt		

## Supplementary Materials

The following supporting information can be downloaded at: https://www.mdpi.com/article/10.3390/polym14122414/s1, Figure S1: ^1^H NMR (D_2_O) spectrum of CS; Figure S2: ^13^C NMR (D_2_O) spectrum of CS; Figure S3: Photographs of CS in a) solid state and b) aqueous solution when the UV irradiation is off or on; Figure S4: a) Fluorescence lifetime curve of CS in aqueous solution; b) Phosphorescence lifetime curve of CS in aqueous solution (λ_exc._ = 365 nm, λ_monitor_ = 460 nm); Figure S5: Fluorescence spectrum of CS/PVA hybrids (λ_exc._ = 980 nm); Figure S6: TEM images of a) water-soluble UCNPs and b) oil-soluble UCNPs; Figure S7: DLS data of a) water-soluble UCNPs and b) oil-soluble UCNPs; Figure S8: a) Emission spectra of water-soluble UCNPs; b) The lifetime curves of water-soluble UCNPs at the corresponding emission wavelength (345 nm, 362 nm, 450 nm, 480 nm, and 650 nm). (λ_exc._ = 980 nm); Figure S9: a) Emission spectra of oil-soluble UCNPs; b) The lifetime curves of oil-soluble UCNPs at the corresponding emission wavelength (345 nm, 362 nm, 450 nm, 480 nm, and 650 nm). (λ_exc._ = 980 nm); Figure S10: Photographs of a) the CS/w-UCNPs/PVA film and b) the CS/o-UCNPs/PVA film under 365 nm excitation and long-lasting luminescence when the light source withdrawn; Figure S11: Photographs of a) the CS /PVA//w-UCNPs film and b) the CS /PVA//o-UCNPs film under 365 nm excitation and long-lasting luminescence when the light source withdrawn; Figure S12: The afterglow intensities of different films when under a) UV and b) NIR excitation; Figure S13: Fluorescence spectra of (a) IR-26, (c) o-UCNPs; The integrated emission intensities of IR-26 and o-UCNPs plotted against the absorbance at 980 nm. The slopes in (b), (d) were obtained by linear fitting; Videos S1–S7. References [38,39] are cited in Supplementary Materials file.
