# Supplementary material for: Ultralong-Lived Up-Conversional Room-Temperature Afterglow Materials with a Polyvinyl Alcohol Substrate
Source: Polymers (Basel). 2022 Jun 14;14(12):2414. doi: 10.3390/polym14122414 (PMC9229245; doi:10.3390/polym14122414)
Supplement: Supplementary file 1 [file polymers-14-02414-s001.zip › polymers-1751220-SM.pdf]

Support Information

Ultralong-lived up-conversional room-temperature afterglow materials with polyvinyl alcohol-substrate

Lulu Zhou[1], Bin Wu[2], Ben Shi[2], Xinyan Zhu[2], Shen Shen[1], Liangliang Zhu[1]*

1. State Key Laboratory of Molecular Engineering of Polymers, Department of Macromolecular Science, Fudan University, Shanghai 200438, China.
2. State Key Laboratory of Molecular Engineering of Polymers, Department of Chemistry, Fudan University, Shanghai 200433, China.

Email: zhuliangliang@fudan.edu.cn

1. EXPERIMENTAL SECTION

General. All reagents were purchased from Aldrich and used without additional purification. ^{1}H NMR and ^{13}C NMR spectra were measured on a Bruker AVANCE III HD spectrometer. The UV-vis absorption spectra were recorded on a Perkin-Elmer Lambda 750 spectrophotometer. The excitation and emission spectra and the lifetime spectra were all recorded on FLS 1000 (Edinburgh Instruments) with a 980nm CW laser (2W). The photoirradiation was carried out with a hand-held 365nm lamp (5W) and a 980nm laser pointer (200mW). Transmission electron microscopy (TEM) was performed on a Tecnai G2 20 TWIN with an accelerating voltage of 200 kV. Dynamic light scattering (DLS) experiments were carried out with Nano-Zeta Potential Analyzer ZS-90.

Synthesis of **coronene tetracarboxylate salt (CS)** (Figure Scheme S1)**.** Perylene (24 mmol), N-ethyl-maleimide (300 mmol), p-hydroxyanisole (12 mmol) and tetra chloranil (91 mmol) were thoroughly mixed and heated at 240 °C with stirring to reflux for 6 hours. The mixture was vacuum filtered through a glass filter and washed with ethanol and dried to give a yellow powder. The powder was transferred to a 100 mL flask, methanol (60 mL) and potassium hydroxide (40 g) were added and the mixture was heated to boiling temperature with stirring. Allow the methanol to boil and continue to reflux overnight at about 130°C. After cooling to room temperature, most methanol was distilled off by rotary evaporation, then the solid was obtained by suction filtration, and finally the solid was rinsed with a small amount of methanol to obtain the final product. ^{1}H NMR (400 MHz, D_2O, 298 K): δ = 9.00 (m, 8H). ^{13}C NMR (400 MHz, D_2O, 298 K): δ = 177.46, 133.49, 128.45, 127.00, 124.86, 124.54, 121.76, 121.51.

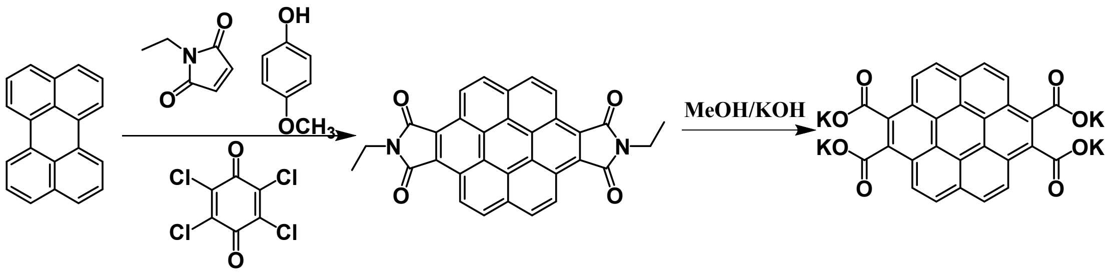

Scheme S1. Synthetic Scheme for the synthesis of **CS**.

Synthesis of **oil-soluble UCNPs.** In a 100 mL three-necked flask, add $YbCl_3·6H_2O$ (1 mmol),

oleic acid (16 mL) and octadecene (24 mL) at the same time, stir and heat to 140 °C and keep for 30 min under vacuum until it becomes clear and transparent with sufficient dehydration and deoxygenation light yellow oily liquid, naturally cooled to below 50 °C. 8 mL of methanol mixed solution dissolved with NaOH (2.5 mmol) and NH_4F (4 mmol) was rapidly added, and then the system was slowly heated to 50 °C and kept for 1 h. After the heat preservation was completed, the mixture was heated to 140 °C under vacuum conditions with sufficient stirring for 30 min. Heated to 290 °C at a heating rate of 10 °C/min under Ar protection and maintained for 100 min. After the system was cooled to room temperature, 5 mL of absolute ethanol was added to separate out the product, and the solid part was filtered after centrifugation at 4500 rpm for 5 min, washed twice with absolute ethanol and cyclohexane, and finally dispersed in cyclohexane to obtain the final product (0.1 M).

Synthesis of **water-soluble UCNPs.** Take out 200 μL of the oil-soluble UCNPs prepared above into 200 μL of absolute ethanol and centrifuge at 5000 rpm, add 200 μL of $CHCl_3$ after suck off the supernatant, then transfer it to a 100 mL eggplant-shaped bottle after sonication, add 5 mL of $CHCl_3$ and 500 μL of 0.1 mM DSPE-mPEG2000 in $CHCl_3$. After all solvent was evaporated, it was placed in an oven overnight. Finally, take out the eggplant-shaped bottle and add 10 mL of 80 °C hot water, and then sonicate at 100 °C until the solution is completely transparent. The solid product obtained after centrifugation at 5000 rpm for 10 min will be added with 200 μL of deionized water to obtain the final product.

Synthesis of **PVA stock solution.** 20 g PVA was weigh into a 25 mL single-necked flask, with 10 mL of deionized water added to it, after stirred uniformly at 90 °C for 1 h, and brought to room temperature, the PVA stock solution has been obtained.

Synthesis of **CS/w-UCNPs/PVA film.** Weigh 1 mg of CS powder, with two drops of deionized water added to dissolve completely in a 5 mL single-neck flask, then 10 μL of 0.1 M water-soluble UCNPs was put into it, and finally add 1 mL of PVA stock solution. After stirring at 60 °C for 1 h, it was evenly spread on a solid quartz cuvette, and then placed this quartz cuvette in a blast oven at 80 °C for 1 h and taken out to obtain CS/w-UCNPs/PVA film.

Synthesis of **CS/o-UCNPs/PVA film.** Weigh 1 mg of CS powder, with two drops of deionized water added to dissolve completely in a 5 mL single-neck flask, then 10 μL of 0.1 M oil-soluble UCNPs was put into it, and finally add 1 mL of PVA stock solution. After stirring at 60 °C for 1 h, it was evenly spread on a solid quartz cuvette, and then placed this quartz cuvette in a blast oven at 80 °C for 1 h and taken out to obtain CS/o-UCNPs/PVA film.

Synthesis of **CS /PVA//w-UCNPs film.** Weigh 1 mg of CS powder, with two drops of deionized water added to dissolve completely in a 5 mL single-neck flask, and then add 1 mL of PVA stock solution in. After stirring at 60 °C for 1 h, it was evenly spread on a solid quartz cuvette, and then placed in a blast oven at 80 °C for 1 h and taken out to obtain CS/PVA film. After that, 1 mL of 1 M water-soluble UCNPs was evenly spread on the surface of CS/PVA film, and then returned to 80 °C blast oven for 1 h to obtain CS/PVA//w-UCNPs film.

Synthesis of **CS /PVA//o-UCNPs film.** Weigh 1 mg of CS powder, with two drops of deionized water added to dissolve completely in a 5 mL single-neck flask, and then add 1 mL of PVA stock solution in. After stirring at 60°C for 1 h, it was evenly spread on a solid quartz cuvette, and then placed in a blast oven at 80 °C for 1 h and taken out to obtain CS/PVA film. After that, 1 mL of 1 μM oil-soluble UCNPs was evenly spread on the surface of CS/PVA film, and then returned to 80 °C blast oven for 1 h to obtain CS/PVA//o-UCNPs film.

2. ADDITIONAL EXPERIMENTAL DATA

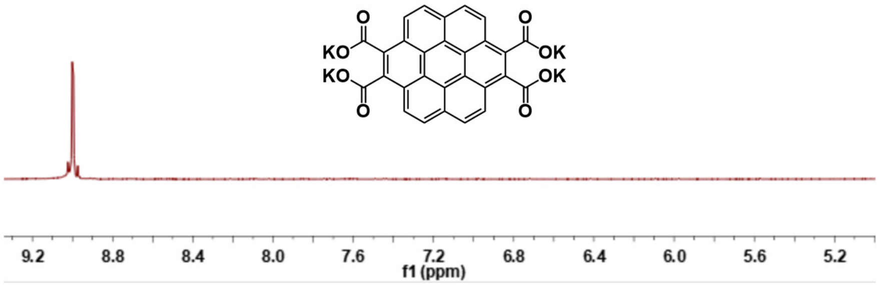

Figure S1. ^1H NMR (D_2O) spectrum of CS.

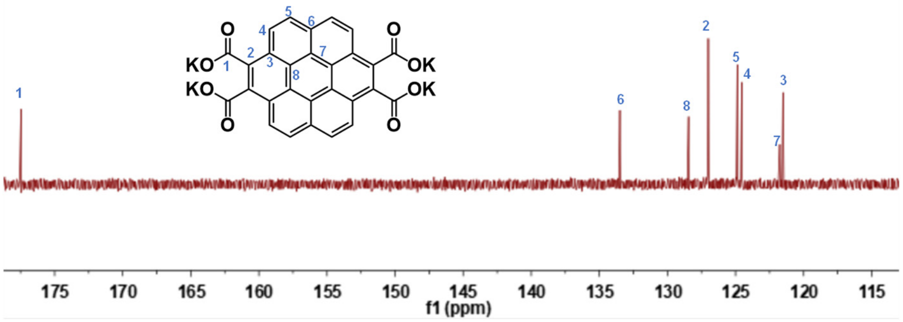

Figure S2. ^{13}C NMR (D_2O) spectrum of CS.

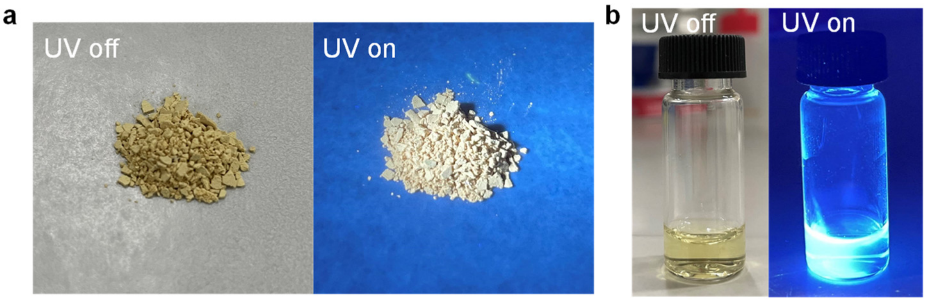

Figure S3. Photographs of CS in a) solid state and b) aqueous solution when the UV irradiation is off or on.

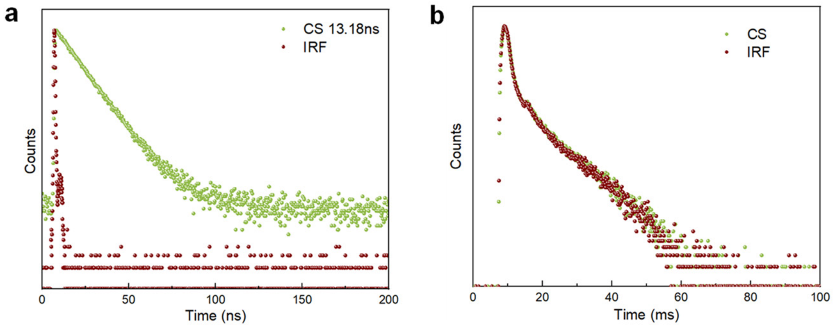

Figure S4. a) Fluorescence lifetime curve of CS in aqueous solution; b) Phosphorescence lifetime curve of CS in aqueous solution ($\lambda_{exc.}$ = 365 nm, $\lambda_{monitor}$ = 460 nm).

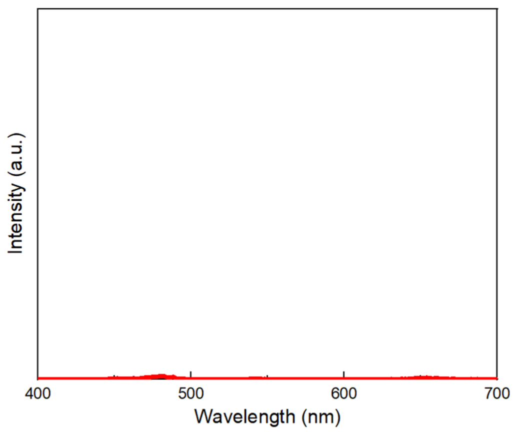

Figure S5. Fluorescence spectrum of CS/PVA hybrids ($\lambda_{exc.}$ = 980 nm).

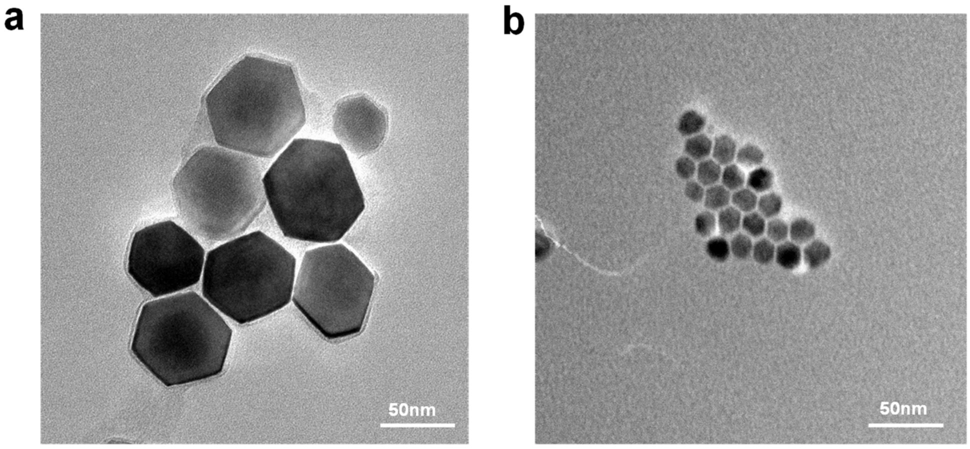

Figure S6. TEM images of a) water-soluble UCNPs and b) oil-soluble UCNPs.

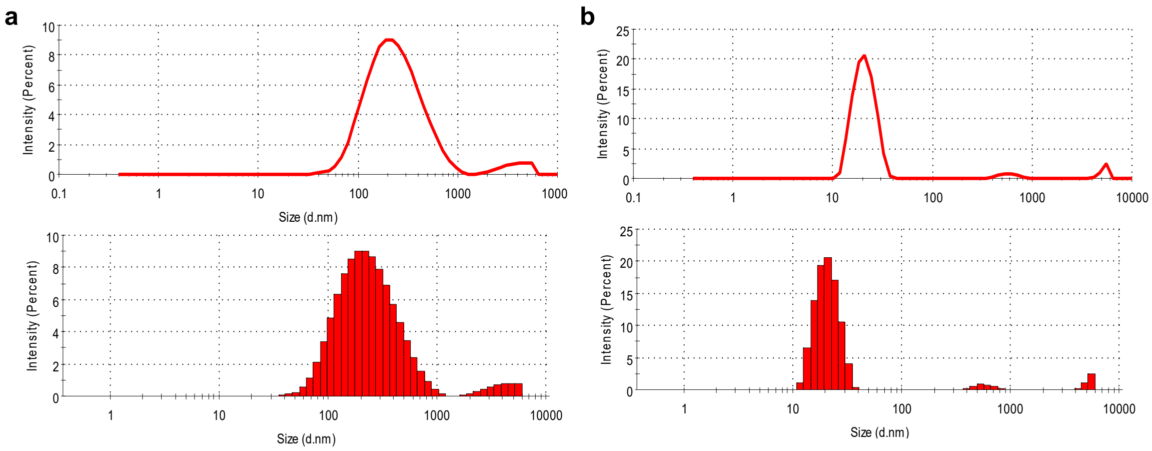

Figure S7. DLS data of a) water-soluble UCNPs and b) oil-soluble UCNPs

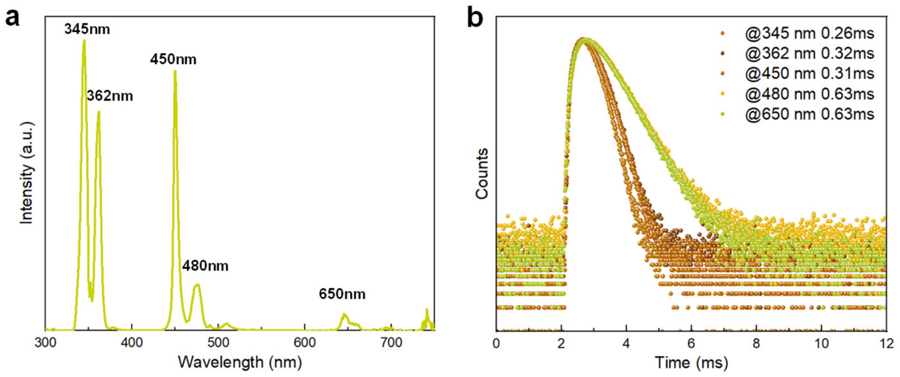

Figure S8. a) Emission spectra of water-soluble UCNPs; b) The lifetime curves of water-soluble UCNPs at the corresponding emission wavelength (345 nm, 362 nm, 450 nm, 480 nm, and 650 nm). ($\lambda_{exc.}$ = 980 nm)

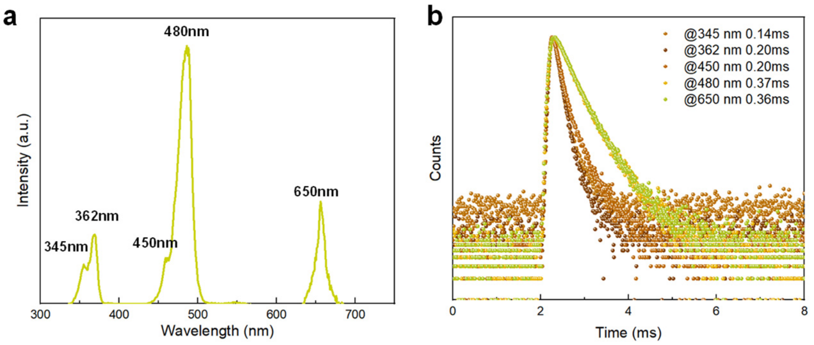

Figure S9. a) Emission spectra of oil-soluble UCNPs; b) The lifetime curves of oil-soluble UCNPs at the corresponding emission wavelength (345 nm, 362 nm, 450 nm, 480 nm, and 650 nm). ($\lambda_{exc.}$ = 980 nm)

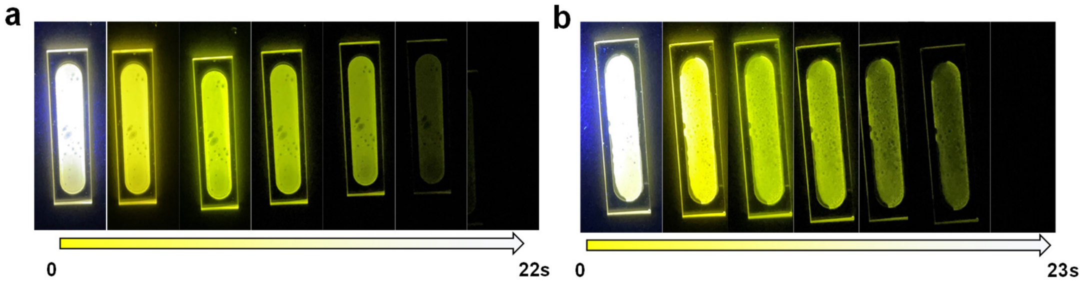

Figure S10. Photographs of a) the CS/w-UCNPs/PVA film and b) the CS/o-UCNPs/PVA film under 365 nm

excitation and long-lasting luminescence when the light source withdrawn.

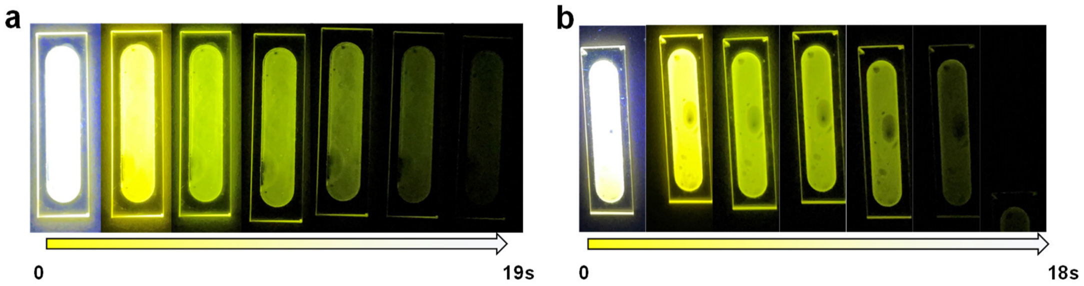

Figure S11. Photographs of a) the CS /PVA//w-UCNPs film and b) the CS /PVA//o-UCNPs film under 365 nm excitation and long-lasting luminescence when the light source withdrawn.

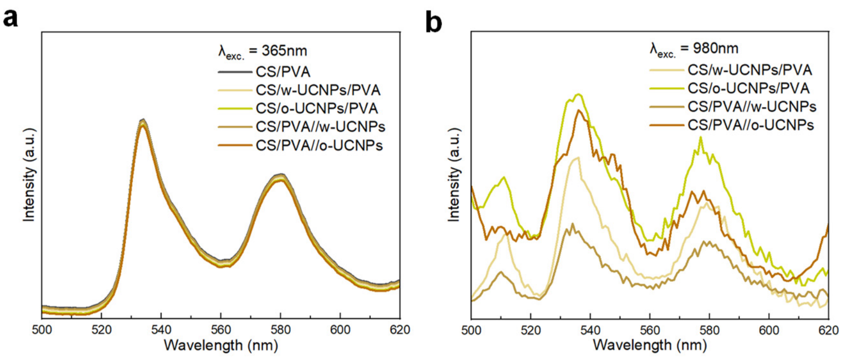

Figure S12. The afterglow intensities of different films when under a) UV and b) NIR excitation.

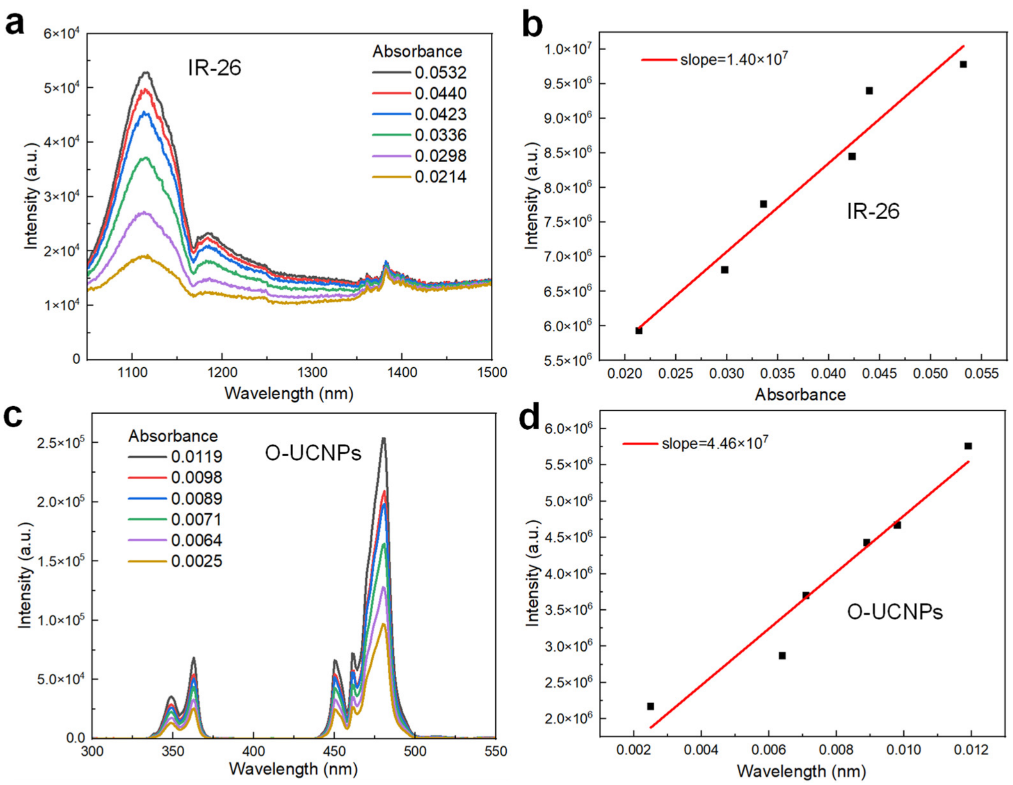

Figure S13. Fluorescence spectra of (a) IR-26, (c) o-UCNPs; The integrated emission intensities of IR-26 and o-UCNPs plotted against the absorbance at 980 nm. The slopes in (b), (d) were obtained by linear fitting.

The QY of the o-UCNPs was measured using a standard dye IR-26 as the reference[1, 2]. The IR-26 and the o-UCNPs were excited by a 980 nm laser using the same power density. The calculation equation was estimated as $Q_{o-UCNPs} = Q_{ref} \times \frac{S_{o-UCNPs}}{S_{ref}} \times \left(\frac{n_{o-UCNPs}}{n_{ref}}\right)^2$, where $Q_{o-UCNPs}$ is the QY of the o-UCNPs, Q_{ref} is the quantum yield of IR-26 (~ 0.05%), and $S_{o-UCNPs}$ is the slope obtained by linear fitting of the integrated emission spectra of o-UCNPs (300 nm ~ 550nm) against the absorbance at 980 nm, S_{ref} is the slope obtained by linear fitting of the integrated emission spectra of IR-26 (1050 nm ~ 1500nm) against the absorbance at 980 nm and $n_{o-UCNPs}$, n_{ref} are the refractive indices of their respective solvents (cyclohexane: 1.42662; and dichloroethane: 1.4167).

References:

1. Fan, Y.; Wang, P.; Lu, Y.; Wang, R.; Zhou, L.; Zheng, X.; Li, X.; Piper, J. A.; Zhang, F., Lifetime-engineered NIR-II nanoparticles unlock multiplexed in vivo imaging. *Nat Nanotechnol* **2018,** *13* (10), 941-946.

2. Zhong, Y.; Ma, Z.; Zhu, S.; Yue, J.; Zhang, M.; Antaris, A. L.; Yuan, J.; Cui, R.; Wan, H.; Zhou, Y.; Wang, W.; Huang, N. F.; Luo, J.; Hu, Z.; Dai, H., Boosting the down-shifting luminescence of rare-earth nanocrystals for biological imaging beyond 1500 nm. *Nat Commun* **2017,** *8* (1), 737.
